# Effect of the Formation of Ultrathin Selective Layers on the Structure and Performance of Thin-Film Composite Chitosan/PAN Membranes for Pervaporation Dehydration

**DOI:** 10.3390/membranes10070153

**Published:** 2020-07-16

**Authors:** Mariia Dmitrenko, Andrey Zolotarev, Tatiana Plisko, Katsiaryna Burts, Vladislav Liamin, Alexandr Bildyukevich, Sergey Ermakov, Anastasia Penkova

**Affiliations:** 1 St. Petersburg State University, 7/9 Universitetskaya nab., 199034 St. Petersburg, Russia; m.dmitrienko@spbu.ru (M.D.); andrey.zolotarev@spbu.ru (A.Z.); lyamin.vlad.322@gmail.com (V.L.); s.ermakov@spbu.ru (S.E.); 2 Institute of Physical Organic Chemistry, National Academy of Sciences of Belarus, 13 Surganov Str., 220072 Minsk, Belarus; plisko.v.tatiana@gmail.com (T.P.); katyaburt@gmail.com (K.B.); uf@ifoch.bas-net.by (A.B.)

**Keywords:** thin-film composite membrane, interfacial polymerization, pervaporation

## Abstract

The aim of the study is to improve the performance of thin-film composite (TFC) membranes with a thin selective layer based on chitosan (CS) via different approaches by: (1) varying the concentration of the CS solution; (2) changing the porosity of substrates from polyacrylonitrile (PAN); (3) deposition of the additional ultrathin layers on the surface of the selective CS layer using interfacial polymerization and layer-by-layer assembly. The developed membranes were characterized by different methods of analyses (SEM and AFM, IR spectroscopy, measuring of water contact angles and porosity). The transport characteristics of the developed TFC membranes were studied in pervaporation separation of isopropanol/water mixtures. It was found that the application of the most porous PAN-4 substrate with combination of formation of an additional polyamide selective layer by interfacial polymerization on the surface of a dense selective CS layer with the subsequent layer-by-layer deposition of five bilayers of poly (sodium 4-styrenesulfonate)/CS polyelectrolyte pair led to the significant improvement of permeance and high selectivity for the entire concentration feed range. Thus, for TFC membrane on the PAN-4 substrate the optimal transport characteristics in pervaporation dehydration of isopropanol (12–90 wt.% water) were achieved: 0.22–1.30 kg/(m^2^h), 99.9 wt.% water in the permeate.

## 1. Introduction

Distillation, low temperature crystallization, adsorption and extraction are common separation methods for liquid mixtures, but these methods, as a rule, are energy-intensive, labor-intensive and have a negative impact on the environment [1,2,3]. In addition, the distillation method cannot separate the azeotropic mixtures of liquid substances without using the additional third agents like benzene or cyclohexane, which may cause an impurity in the product. Membrane processes are an alternative to traditional separation methods. Pervaporation is one of the most efficient membrane methods for the separation of low molecular weight liquid mixtures, which is widely used for the dehydration of alcohols and other organic substances [4,5,6,7,8,9] and characterized by high efficiency with low energy consumption, a compact equipment, environmental friendliness and the ability of the automation [10]. Pervaporation is also a promising and perspective technology for the separation of azeotropic mixtures without application of additional reagents.

Various polymeric and inorganic membranes have been developed for the pervaporation dehydration of alcohols [4,5,6,11,12,13,14,15,16,17]. Polymeric membranes are more attractive due to the fabrication simplicity, flexibility and cost-effectiveness compared to inorganic membranes. Green water-soluble polymers, such as polyvinyl alcohol (PVA), chitosan (CS), alginates (Alg) are the widely applied and popular membrane materials for pervaporation dehydration of alcohols because of environmental friendliness and high selectivity to water [14]. Today, CS is likely the most explored biopolymer in the membrane fabrication due to its biocompatibility. However, membranes based on this polymer require additional cross-linking as they swell uncontrollably in an aqueous medium reducing mechanical and thermal instability [18]. And it is also worth noting that cross-linking of CS membranes by various ways, as a rule, leads to a decrease in membrane permeability. The low permeability and mechanical strength of pervaporation CS membranes are limiting factors for the widespread adoption of these membranes in industrial processes of alcohol dehydration.

One of the relevant ways to obtain the high-performance membranes is to apply a thin non-porous selective polymeric layer on ultra- or microfiltration membranes (substrates). This type of membrane is known as thin-film composite membrane (TFC). It is very important to select the suitable substrate and the polymer layer that provides selective separation and high permeability (due to the small thickness of the layer). The substrate should have (1) a highly porous structure of the upper selective layer and the substructure to minimize resistance to mass transfer; (2) a thin defect-free upper selective layer with a small pore diameter and a narrow pore size distribution to prevent the penetration of polymer solution into the substrate and for the formation of selective polymer layer without defects [19,20]; (3) the absence of large pores (macrovoids) in the substrate substructure to ensure high mechanical strength. The non-porous and defect-free polymer selective layer should be as thin as possible to obtain high membrane separation selectivity, what can be achieved by varying the concentration of the polymer solution used for thin selective layer formation. One of the key advantages of TFC membrane preparation is that a thin selective layer and a substrate can be optimized independently of each other to achieve the desired membrane morphology and to maximize separation efficiency.

The achievement of high flux and selectivity of membranes for hydrophilic pervaporation is also possible by creating a membrane with a hierarchical structure with the deposition of additional ultrathin layers by interfacial polymerization (IP) and layer-by-layer (LbL) assembly on the membrane surface, which can also contribute to healing of the thin selective polymer layer defects of the TFC membrane and to change membrane surface properties.

Interfacial polymerization is a method of synthesizing an ultrathin functional polymer layer at the phase boundary during the reaction between two immiscible solutions, such as an aqueous solution of an amine and a solution of an acyl chloride in an organic solvent [19,20]. Membranes prepared by IP can be cost effective and promising for industrial production and application [21]. Recently, membranes created by the IP method have found application in pervaporation [1,3,22,23,24,25,26,27,28,29]. It was shown that using IP technology for the manufacture of membranes it was possible to achieve improved transport characteristics of the membrane in pervaporation separation of a water/ethanol mixture (permeation flux up to 13.9 kg/(m^2^h) and 4491 separation factor) due to the formation of ultrathin heterogeneous polyamide selective layers [22]. The development of pervaporation membranes with IP method is a complex technological and scientific task due to the large number of parameters that need to be controlled (substrate structure, composition and method of forming the PA layer, drying and cross-linking conditions, etc.). The number of papers devoted to the development of pervaporation TFC membranes by interfacial polymerization is relatively few, which is associated with the high difficulty of the problem, the need to control a large number of parameters, and the difficulty of creating an ultrathin defect-free selective layer on the surface of a porous substrate.

One of the modern methods for controlling the surface properties of membranes is the layer-by-layer assembly (LbL) for the deposition of nanosized polyelectrolyte (PEL) layers on the surface of a membrane [30,31]. This technology consists of sequential alternating deposition of polycations and polyanions on the membrane surface, which allows controlling the thickness and properties of the membrane surface (for example, hydrophilicity, roughness, surface charge, etc.) varying conditions such as types and the number of PEL layers, PEL ionic strength, pH of PEL, etc. One of the wide spread approaches to carry out the surface modification by LbL is via dip-LbL method. In our previous studies [15,32,33] it was demonstrated that changing various parameters of LbL assembly such as the selected pair of polycation–polyanion, the order of their deposition, the number of cycles, and additional bulk modification of membranes significantly affected the transport characteristics of the membranes.

Thus, this work is devoted to the improvement of the pervaporation isopropanol dehydration performance of TFC membranes with a thin selective layer based on chitosan by several approaches: (1) varying concentration of the CS solution; (2) varying the porosity of PAN membranes (substrates); and (3) deposition of the additional ultrathin layers on the surface of the chitosan layer by interfacial polymerization and the layer-by-layer assembly. The paper performs step-by-step development and characterization by different methods of thin-film composite membranes with hierarchical structure starting from the anisotropic porous membrane-substrate and creation of selective layers via physical adsorption, interfacial polymerization and layer-by-layer assembly techniques.

## 2. Materials and Methods

### 2.1. Materials

Polyacrylonitrile (PAN) ultrafiltration membranes developed at the Institute of Physical Organic Chemistry of the National Academy of Sciences of Belarus (Minsk, Belarus) were used as porous membrane-substrates for the formation of TFC membranes. Poly(acrylonitrile-co-methyl acrylate) copolymer (ratio of acrylonitrile and methyl acrylate monomer units: 93.6:6.4, M_w_ = 76,000 g/mol, M_w_/M_n_ = 2.88; η_sp_ = 0.76, Haihang Industry Co., Ltd., Jinan, China) was used as the membrane material for the preparation of casting solutions for ultrafiltration PAN membranes (substrates) via the non-solvent induced phase inversion (NIPS). N,N-dimethylformamide (DMF, reagent grade, Khimmed, Moscow, Russia) was used as a solvent.

Chitosan (CS) (with medium molecular weight, “Sigma-Aldrich Co.”, St. Louis, MO, USA) was applied as a polymer material for the formation of a thin selective sublayer (the first dense layer) on ultrafiltration substrates. The maleic acid (MA, “Sigma-Aldrich Co.”, St. Louis, MO, USA) was used as cross-linking agent to stabilize the thin dense CS layer. MA was applied without additional treatment.

Triethylenetetramine (TETA, ≥96%, “BASF”, Ludwigshafen, Germany) as the amine component and trimesoyl chloride (TMC, ≥99%, “Sigma-Aldrich Co.”, St. Louis, MO, USA) as an acyl component were used for the formation of a second (upper) ultrathin selective layer on the TFC membrane by the interfacial polymerization technique. Distilled water was used to prepare TETA solutions, and «NEFRAS S2» (80/120, “Vershina” LLC, St. Petersburg, Russia) was used as an organic solvent for TMC. «NEFRAS S2» is a mixed type gasoline solvent, which contains not more than 50% hydrocarbons of each group; low boiling fraction of dearomatized gasoline of catalytic reforming.

Isopropanol (chemically pure, “Vecton”, St. Petersburg, Russia) and distilled water without additional treatment were used in pervaporation experiments.

Bovine serum albumin (BSA, M = 68,000 g/mol, “PanReac AppliChem”, Moscow, Russia) with a concentration of 0.5 g/L in phosphate buffer solution at pH = 7, aqueous solution of PVP K-30 (Mn = 40,000 g/mol, “Fluka”, Munich, Germany) and PVP K-15 (Mn = 10,000 g/mol, “Fluka”, Munich, Germany) with a concentration of 3 g/L were used as model solutions for rejection tests in ultrafiltration.

Poly (allylamine hydrochloride) (PAH, Mw = 50,000 g/mol, “Sigma-Aldrich Co.”, St. Louis, MO, USA) and chitosan (with medium molecular weight, “Sigma-Aldrich Co.”, St. Louis, MO, USA) were used as cationic polyelectrolytes, and poly (sodium 4-styrenesulfonate) (PSS, “Sigma-Aldrich Co.”, St. Louis, MO, USA) was used as an anionic polyelectrolyte in layer-by-layer assembly.

### 2.2. Membrane Preparation

#### 2.2.1. Porous PAN Membrane (Substrate) Preparation

Porous anisotropic membranes (substrates) were prepared via non-solvent induced phase separation (NIPS) using a polyester non-woven fabric (JSC «Komiteks», Syvtyvkar, Komi Republic, Russia) as a mechanical support. To obtain flat porous PAN membranes by NIPS, a polymer solution (at 20 °C) was cast on a polyester non-woven support fixed to a glass plate using a casting blade (the thickness of the polymer layer was 160 μm). A glass plate with a layer of casting solution on a polyester non-woven support was immersed in a coagulation bath with distilled water at 25 °C. Four porous membranes from PAN were prepared marked as PAN-2, PAN-3, PAN-4, PAN-5 which feature different structure, pore size, porosity of the selective layer and performance. The compositions of the casting solution were selected according to [34]. PAN-2 membrane was prepared from 16 wt.% PAN casting solution in DMF, PAN-3—from 15 wt.% PAN solution in DMF. To obtain PAN-4 and PAN-5 membranes 14 wt.% and 13 wt.% PAN casting solutions were used respectively. The obtained ultrafiltration membranes were impregnated by 10% glycerol aqueous solution to prevent the capillary contraction of pores of the selective layer. The membranes were dried at room temperature.

#### 2.2.2. Formation of the First Dense Selective Layer on Porous PAN Membranes (Substrates)

The method of physical adsorption was used to form the first dense selective CS layer on porous membranes (substrates). TFC membranes with a thin selective layer based on chitosan were prepared by deposition of two layers of a chitosan solution on ultrafiltration PAN substrates with different porosity, followed by evaporation of the solvent for 24 h at 25 °C. For chitosan-based membranes 1 and 0.5 wt.% polymer solutions were prepared in a 1 wt.% solution of acetic acid in water with vigorous stirring and addition of maleic acid (35 wt.% MA with respect to the CS weight). After evaporation of the solvent CS membranes were heated at 110 °C for 120 min.

#### 2.2.3. Formation of the Second (Upper) Layer by Interfacial Polymerization

**Figure 1:**
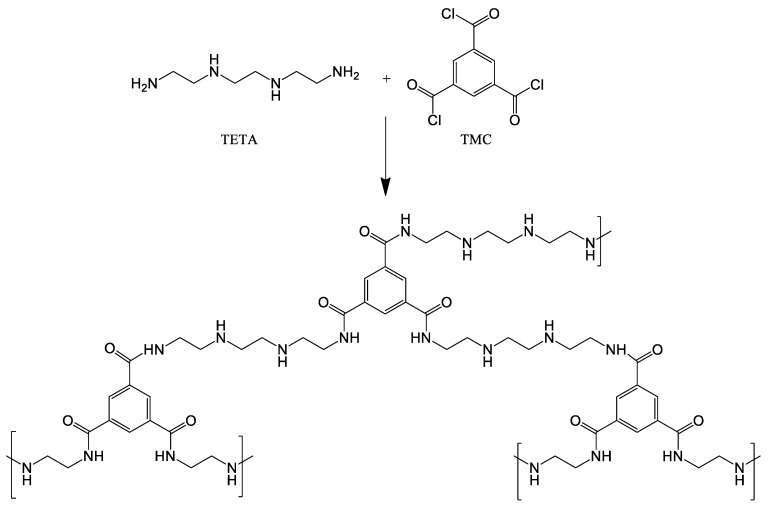
Reaction between TETA and TMC in the interfacial polymerization.

0.1 wt.% aqueous TETA solution and 0.05 wt.% TMC solution in NEFRAS S2 were used to obtain ultrathin selective layer via interfacial polymerization technique. IP was carried out according to the following procedure: the membranes were soaked in TETA solution for 10 s, thereafter, the excess of an aqueous amine solution was removed using filter paper and the membrane was dried at ambient temperature for 15 min, then the membrane was immersed in a TMC solution for 10 s (Figure 1). Thereafter the membrane was left at room temperature until NEFRAS S2 evaporated completely. To remove residual monomers the membrane was immersed in ethanol for 10 min and then dried to evaporate the alcohol in oven at 30 °C for 24 h.

### 2.3. Membrane Investigation Methods

#### 2.3.1. Scanning Electron Microscopy (SEM)

SEM micrographs of the cross-section and the selective surface structure of CS-based TFC membranes and PAN substrates were obtained using Zeiss Merlin SEM (Carl Zeiss SMT, Oberhochen, Germany). The cross-section of samples was obtained by submerging the sample in liquid nitrogen for 10–20 s and subsequent splitting perpendicular to the surface. SEM images were obtained at a voltage of 1 kV.

#### 2.3.2. Atomic Force Microscopy (AFM)

The topography of the selective layer surface of CS-based TFC membranes and PAN porous anisotropic membrane-substrates were studied on an NT-MDT nTegra Maximus atomic force microscope with standard silicon cantilevers with a rigidity of 15 N∙m^−1^ (“NT-MDT”, Moscow, Russia) in tapping mode.

#### 2.3.3. The Standard Porosimetry Method

The porosity of the porous PAN membrane-substrates was determined by the standard porosimetry method on a Porosimeter 3.1 instrument (Porotech Ltd., Ottawa, ON, Canada) at 30 °C. n-Octane was used as the reference liquid.

#### 2.3.4. Ultrafiltration

**Figure 2:**
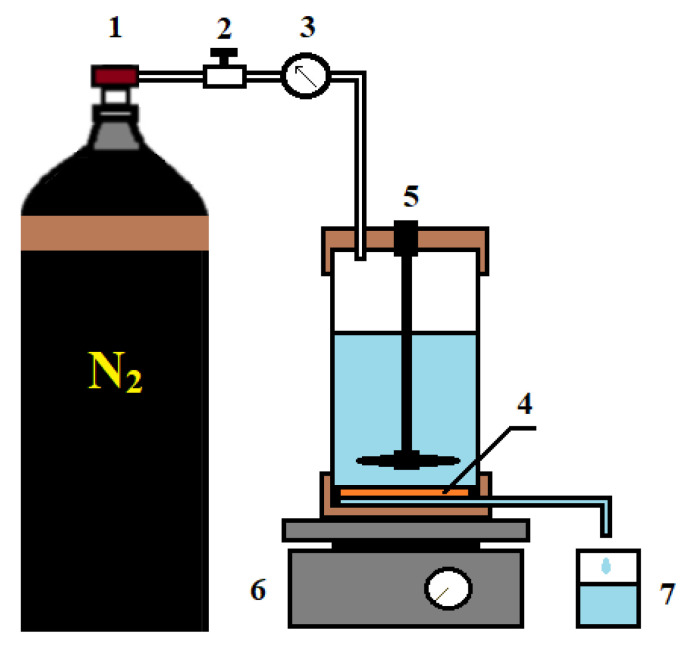
Ultrafiltration setup: 1—nitrogen tank, 2—pressure regulator, 3—manometer, 4—membrane, 5—stirred ultrafiltration cell, 6—magnetic stirrer, 7—container for permeate.

PAN porous substrates (ultrafiltration membranes) used for the preparation of supported CS-based membranes were studied in ultrafiltration to evaluate their performance (pure water flux, flux of BSA or PVP solutions, rejection coefficients of BSA or PVP, flux recovery ratio). The measurements were carried out on a laboratory setup (stirred ultrafiltration cell) at room temperature (25 °C) with a transmembrane pressure of 1 bar and a stirring speed of 250–300 rpm (Figure 2). BSA solution (0.5 g/L) prepared in a phosphate buffer solution (pH = 7) was used as a protein foulant to study antifouling performance.

The flux was determined as follows: ultrafiltration of pure water at 1 bar and room temperature was carried out for 30 min to reach the stationary ultrafiltration mode of the membrane and to wash it from glycerol. Thereafter, pure water flux was determined. Then a solution of BSA or PVP was placed into the cell and filtered for 30 min under the same conditions. Thereafter the BSA or PVP solution flux was measured. The flux *J* (L/m^2^h) was calculated according to the equation:

(1) $$J=\frac{V}{A\times t},$$

where *V* (L) is the volume of liquid passing through the membrane (permeate), *A* (m^2^) is the membrane area and *t* (h) is the time of ultrafiltration.

The BSA concentration in the feed solution and permeate was determined using SF-102 spectrophotometer at a wavelength of 280 nm. The PVP concentration was determined using LIR-2 interferometer («Zagorsk optical and mechanical plant», Sergiev Posad, Russia). The rejection coefficient *R* (%) was calculated according to the equation:

(2) $$R=\left(1-\frac{Cp}{Cf}\right)\times 100\%,$$

where Cp (g/L) is the BSA or PVP concentration in permeate, Cf (g/L) is the BSA or PVP concentration in the feed solution.

The pure water flux was measured 30 min after ultrafiltration to assess the antifouling properties of the membranes in ultrafiltration of BSA solution. Then the BSA solution was filtered for 1 h. Thereafter water was again passed through the membrane for 30 min and pure water flux was measured again. The flux recovery ratio *FRR* (%) was calculated according to the equation:

(3) $$FRR=\left(\frac{J}{J_{0}}\right)\times 100\%,$$

where *J* (L/(m^2^h)) is the pure water flux after BSA solution ultrafiltration and *J*_0_ is the initial pure water flux.

#### 2.3.5. Fourier-Transform Infrared Spectroscopy (IR Spectroscopy)

IR spectra of the selective layer surface of TFC membranes were obtained on a BRUKER-TENSOR 27 IR Fourier spectrometer (Bruker Optik GmbH, Billerica, MA, USA) in the range of 700–4000 cm^−1^ at 25 °C to confirm the formation of polyamide upper layer by IP method.

#### 2.3.6. Pervaporation

**Figure 3:**
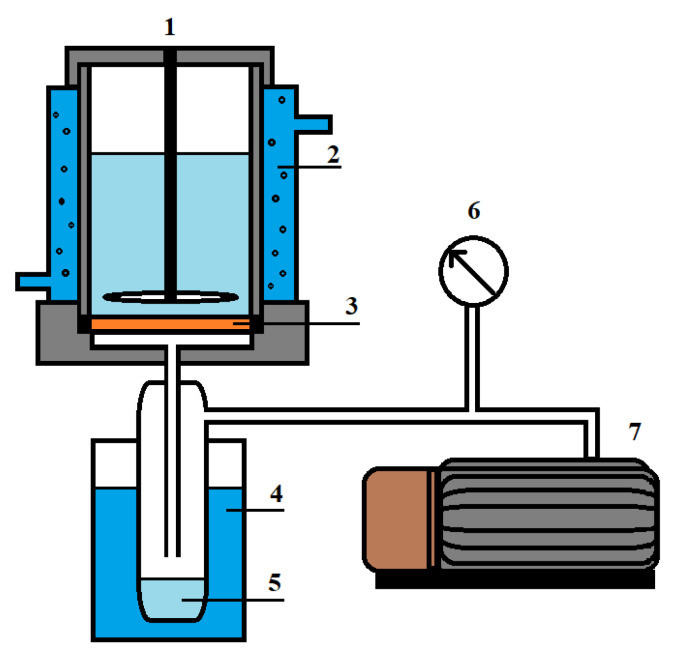
Pervaporation setup: 1—pervaporation cell, 2—temperature controller, 3—membrane, 4—trap cooled by liquid nitrogen, 5—permeate; 6—manometer and 7—vacuum pump using to control the pressure.

The transport characteristics of the developed membranes were studied by vacuum pervaporation in a steady-state laboratory setup (Figure 3) for the separation of the isopropanol/water mixture in a wide concentration range (12–100 wt.% water in the feed) and at various temperatures (28, 35 and 50 °C). The pervaporation condition was the pressure < 0.01 mmHg in the submembrane space.

The composition of the feed and the permeate was determined using SHIMADZU GC-2010 gas chromatograph. The permeation flux *J* (kg/(m^2^h) was calculated according to the equation:

(4) $$J=\frac{W}{A\times t},$$

where *W* (kg) is the permeate weight passing through the membrane, *A* (m^2^) is the membrane area and *t* (h) is the measurement time.

The permeance (*P*/*l*) (component flux normalized for the driving force) was calculated according to the equation:

(5) $$\frac{P}{l}=\frac{j_{i}}{p_{i_{f}}-p_{i_{p}}},$$

where *j_i_* is the partial flux of the *i* component, $p_{i_{f}}$ and $p_{i_{p}}$ are the component vapor pressures of the feed and the permeate, respectively, *l* is the membrane thickness.

The selectivity (*β*) which is the ratio of component permeances was calculated according to the equation:

(6) $$\beta =\frac{P_{i}/l}{P_{j}/l},$$

where *P_i_*/*l* (GPU) is the water permeance and *Pj*/*l* (GPU) is the isopropanol permeance.

The membrane flash index (*MFLI*) was calculated according to the equation [35]:

(7) $$MFLI=\frac{y_{i}^{PV}}{y_{i}^{VLE}},$$

where $y_{i}^{PV}$ is the component concentration in the permeate (wt.%), $y_{i}^{VLE}$ is the component vapor concentration (the equilibrium distillation value) (wt.%).

#### 2.3.7. Contact Angle Determination

Water contact angles for the selective layer surface of PAN ultrafiltration membranes (substrates) were determined by the attached bubble technique using the LK-1 goniometer (“Otkrytaya nauka”, Krasnogorsk, Russia) in the “membrane surface-water-air bubble” system to study the changes of surface properties. Water contact angles for CS-based TFC membranes were determined by sessile drop method using LK-1 goniometer. The measurements were carried out only from the side of the selective layer to study the surface characteristics of the membranes.

#### 2.3.8. Layer-by-Layer Assembly

Membranes with polyelectrolyte nanolayers were prepared using the ND Multi Axis Dip ND-3D 11/5 robot (Nadetech, Navarra, Spain), which had a wide range of membrane immersion rates in solutions (1–2000 mm/min) providing high reproducibility of the deposited layer thickness. The deposition of polyelectrolyte (PEL) layer was carried out as follows: a membrane fixed on special substrate was immersed in a polyanion solution of PSS (10^−2^ mol/L) for 10 min, then in water to wash and thereafter in a polycation solution of PAH (10^−2^ mol/L, pH = 4) or CS (4.7 wt.%) for 10 min, then membrane was washed again. Thus, one bilayer of PEL was created on the surface. In this work the optimal number of cycles (depositions) is five bilayers.

## 3. Results

Various conditions of the formation of a chitosan selective layer on the surface of anisotropic porous PAN membranes (substrates) were studied. The study of the influence of the PAN substrate porosity on the structure and performance of CS-based TFC membranes was carried out. The effect of the concentration of CS solution on the structure and performance of TFC membranes was investigated. The effect of the additional ultrathin selective layer formation via IP and layer-by-layer assembly methods was studied. Section 3.1 is devoted to the characterization of PAN substrates, and Section 3.2 covers the characterization of TFC membranes obtained by deposition of a selective layer from CS on the PAN substrates with or without ultrathin selective layers formed via IP method and layer-by-layer assembly.

### 3.1. Characterization of Porous PAN Substrates

**Figure 4:**
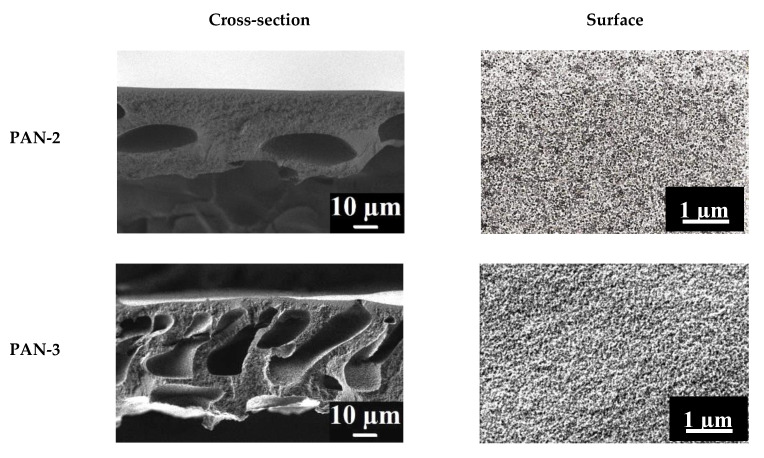

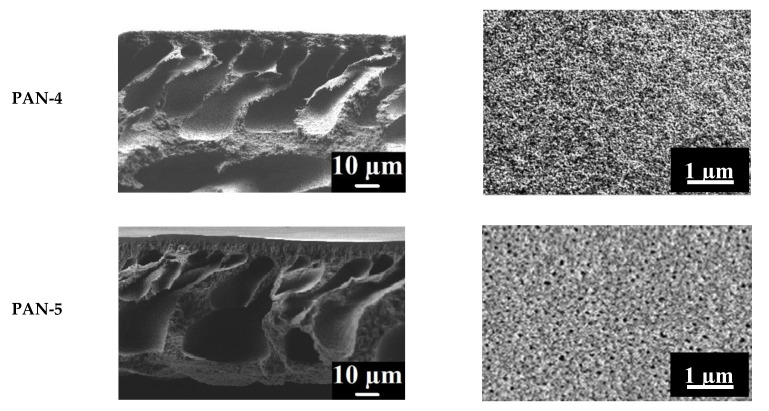
SEM micrographs of the cross-sections and surfaces of the selective layers of the porous PAN membranes (substrates).

The morphology of ultrafiltration membranes (substrates) was studied by AFM and SEM to assess the effect of the structure of the prepared PAN substrates on the properties of TFC CS-based membranes. SEM micrographs of the cross-section and the surface of the selective layer of the PAN substrates are shown in Figure 4.

It was shown that all studied porous PAN membranes featured anisotropic structure typical of membranes prepared by NIPS with a thin selective layer on the porous membrane matrix pierced by macrovoids (Figure 4). The following differences are observed in SEM cross-sectional micrographs of PAN substrates: the size of the macrovoids of the cross-section increases in the series PAN-2 < PAN-3 < PAN-4 < PAN-5. The cross-section of the PAN-2 substrate has a uniform and dense structure with uniformly distributed voids in its structure. The cross-section of the PAN-3,4,5 substrates have a characteristic structure (“finger-shaped” pores), which is typical for high-performance ultrafiltration membranes. The pore distribution of PAN-4 and PAN-3 substrates are comparable to each other in the internal pore volume, while in PAN-5 substrate macrovoids are wider.

SEM surface microphotographs of the PAN membranes demonstrate that the pore size and porosity of the surface of the selective layer of membranes increase in the series: PAN 2 < PAN 3 < PAN 4 < PAN 5 (Figure 4). The surfaces of the PAN-3 and PAN-4 membranes have rougher structure compared to PAN-2. The surface of the PAN-5 substrate has the most compacted structure of the selective layer with large pores on the surface.

**Figure 5:**
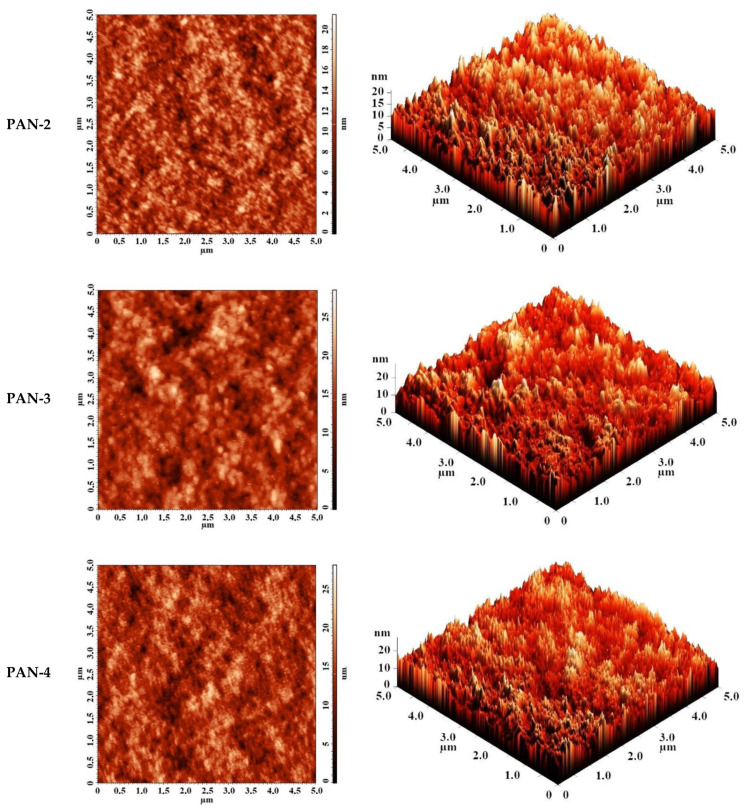

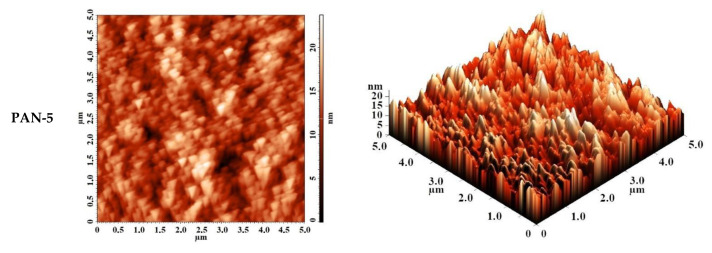
AFM images of the surfaces of the selective layers of porous PAN membranes (substrate).

The surface topography of PAN substrates was studied by AFM. AFM images of PAN substrates with a scan size 5 × 5 µm are shown in Figure 5. The surface of the selective layer features the typical nodular structure for membranes prepared via NIPS. The size of nodules and valleys between them was found to be bigger for PAN-5 membrane increasing as follows: PAN-2 < PAN-3 < PAN-4 < PAN-5.

The roughness characteristics (root-mean-square roughness (Rq) and average surface roughness (Ra)) of ultrafiltration PAN-2,3,4,5 membranes were calculated based on the obtained AFM images and presented in Table 1.

It was shown that the smallest average roughness was observed for the PAN-2 substrate (2 nm), all other substrates PAN-3,4,5 had a slightly higher roughness (~2.5–2.6 nm), which was almost similar to each other. In general, the studied PAN membranes had a low degree of surface roughness of the selective layer.

It was found that the water contact angle for all PAN substrates was equal to 41° ± 2°, which was due to the similar roughness of the selective layer with the same membrane-forming polymer (Table 1).

Analysis of the pure water and PVP K-30 solution flux, PVP K-30 rejection coefficient of the membranes (substrates) suggests a significant difference in pore size and degree of porosity of the studied substrate selective layer (Table 2).

PAN-2 membrane is characterized by the lowest pure water and PVP K-30 solution flux. The PVP K-30 rejection coefficient for PAN-2 membrane is more than 99% due to the high molecular weight of PVP K-30 and small pore size of the selective layer. For a more detailed investigation PVP K-15 (with a lower molecular weight Mn = 10,000 g/mol) solution with a concentration of 3 g/L was also used in ultrafiltration experiments for PAN-2 membrane. It was found, that the flux was equal to 14–15 L/(m^2^h) and the rejection coefficient was also >99%. Thus, it was shown that the PAN-2 membrane is characterized by the smallest pore size of the selective layer (molecular weight cut-off—10 kDa). It is known that the membrane rejection coefficient depends on the pore size of the selective layer and the interaction of the solute molecules with the membrane material, and pure water flux is a characteristic of the pore size and the degree of porosity of the selective membrane layer. Based on the analysis of permeation flux values and PVP rejection coefficient, it was shown that an increase of the average pore size and degree of porosity of the selective layer of the studied porous membranes (substrates) occurred in the following series: PAN-2 < PAN-3 < PAN-4 < PAN-5 (Table 2).

The performance of PAN-2,3,4,5 membranes was also evaluated in the ultrafiltration (UF) of BSA solution at a constant pressure of 1 bar and room temperature. Transport properties (pure water and BSA solution flux, rejection coefficient (R), flux recovery ratio (FRR)) of PAN substrates are presented in Table 2. The total porosity of the substrates was also measured and presented in Table 2. It is known that the porosity of the membrane matrix makes the main contribution to the total membrane porosity, while the permeation flux and selectivity of ultrafiltration membranes are determined by the porous structure and thickness of the selective layer. At the same time the porosity of the selective layer makes a small contribution to the total porosity, therefore the total porosity rather serves as a characteristic of the membrane matrix porosity. However, an increase in the total porosity of the membranes in the series PAN-2 < PAN-3 < PAN-4 < PAN-5 indicates a decrease in membrane matrix mass transfer resistance in membrane separation processes. It was shown that all studied ultrafiltration PAN membranes were characterized by a high BSA rejection coefficient (R ≥ 95%). It was found that BSA solution flux increased in the series PAN-2 < PAN-3 < PAN-4 < PAN-5 (Table 2), what was consistent with the data of the total porosity, SEM, AFM and transport properties of the membranes in ultrafiltration of the PVP K-30 solution. It should be noted that due to the high molecular weight of BSA and its strong interaction with the membrane material (PAN), there are no differences in BSA rejection coefficient for the studied PAN membranes despite differences in pore size and degree of porosity. However, FFR values are an indirect indicator of differences in pore size (Table 2). As it is known, membrane antifouling performance depends on the membrane material, contact angle, degree of roughness, surface charge and pore size of the selective membrane layer [35]. It was shown that in the case of the studied PAN-2,3,4,5 membranes, membrane material (hence the surface charge), the contact angle, degree of roughness were almost similar. It was reported that ultrafiltration membranes prepared from poly(acrylonitrile-co-methyl acrylate) copolymer demonstrate negative zeta-potential of the selective layer surface at pH = 7 [36].

It is worth mentioning that high FRR values for all studied PAN membranes are due to the negative zeta-potential of the surface of the selective layer and negative charge of BSA macromolecules at pH = 7 which yields the efficient electrostatic repulsion of protein macromolecules from membrane surface [37,38,39].

The differences in the flux recovery ratio are primarily associated with different pore sizes of the selective layer. It is known that membranes with a large pore size are more prone to fouling during ultrafiltration and characterized by lower FRR values. Thus, membranes with a small pore size (PAN-2 and PAN-3) are characterized by high FRR values, and the large-pore membranes PAN-4 and PAN-5 show smaller but close FRR values equal to about 78–79%.

### 3.2. Characterization of TFC CS-Based Membranes

**Figure 6:**
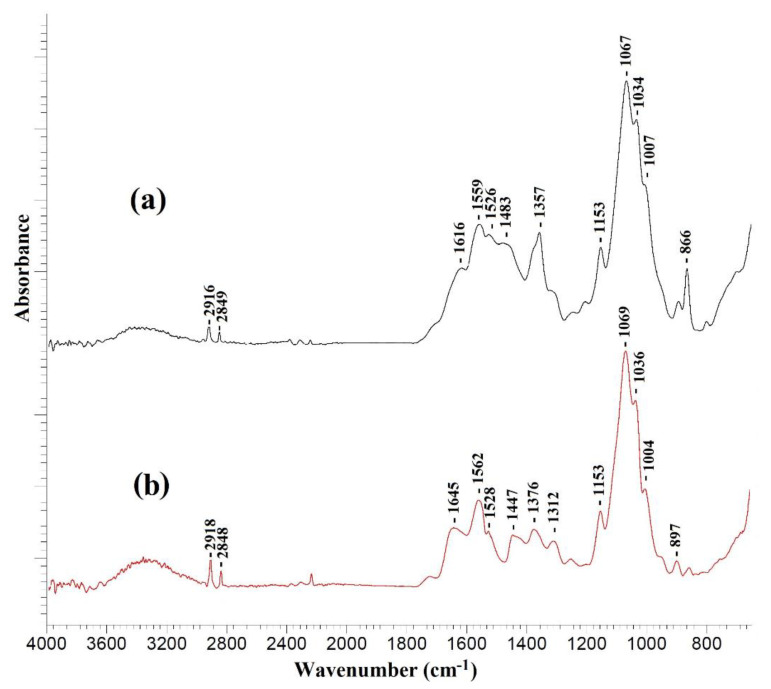
IR spectra of (**a**) CS/PAN-4 and (**b**) CS/PAN-4 with polyamide layer formed via IP.

This section presents the study using various physicochemical methods of TFC membranes with a thin selective layer based on CS deposited on PAN-2,3,4,5 substrates without and with the ultrathin polyamide selective layer formed via IP. To explain the effect of the substrates and mass transfer through the selective CS layer, substrates of the same series were selected, which differ in their porous structure and pure water flux (Table 2). The formation of the polyamide layer as a result of the reaction between TETA and TMC during interfacial polymerization was confirmed by IR spectroscopy (Figure 6).

The IR spectra of all investigated TFC membranes with a selective chitosan layer with or without ultrathin polyamide selective layer were almost identical. Figure 6 demonstrates as example the IR spectra of a TFC membrane obtained on a PAN-4 substrate. It should be noted that formation of a very thin selective polyamide layer on the surface of a cross-linked chitosan layer by IP may lead to the overlapping of the peaks of the chitosan cross-linked by maleic acid and the polyamide layer in the spectrum obtained by the attenuated total reflectance (ATR) method, as evidenced by the large width of the peaks in the range of 1800–1300 cm^−1^.

The occurrence of amide bonds (-CONH-) confirms the formation of a polyamide layer as a result of IP using TETA and TMC and is associated with the following spectral changes: the peak intensity increases in the range of 3400–3200 cm^−1^ and a shift of the peak from 1559 to 1562 cm^−1^ indicates the stretching vibrations of the N-H bond of the amide group [40], as well as the appearance of an intense peak at 1645 cm^−1^ and a wide peak at 1447 cm^−1^, which correspond to stretching vibrations of C=O and C-N bonds of amide (-CONH-) group [40,41].

To study the changes in the hydrophilic-hydrophobic properties of the selective layer surface of TFC CS/PAN membranes after the formation of a thin selective polyamide layer via IP the water contact angles were measured using the sessile drop method. The obtained values of the contact angles of water are presented in Table 3.

As follows from the data in Table 3, the contact angles for all studied CS/PAN membranes were in the range 72–77°, and after IP the contact angles decreased to 59–68° indicating the hydrophilization of the surface of the selective layer, which was due to the excess of a more hydrophilic TETA compared to TMC during IP. This effect was also considered in [42].

**Figure 7:**
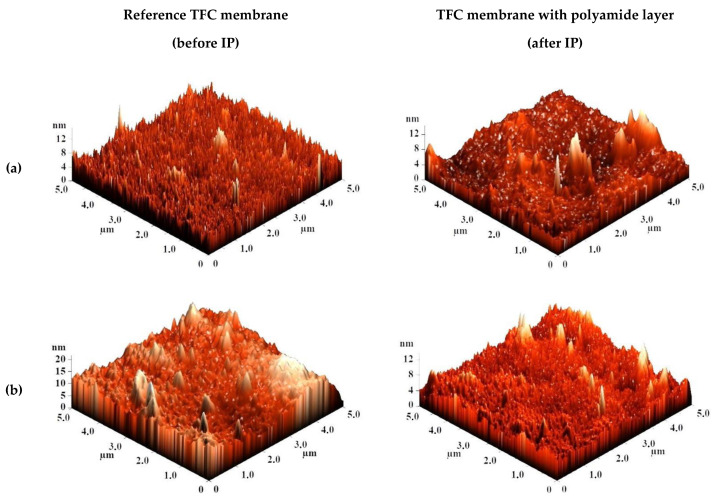

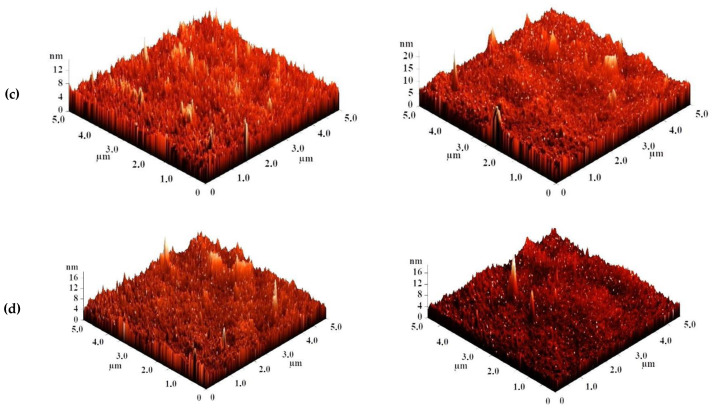
AFM images of TFC membranes (**a**) CS/PAN-2, (**b**) CS/PAN-3, (**c**) CS/PAN-4, (**d**) CS/PAN-5 without and with polyamide layer formed by IP.

The surface topography of the CS-based membranes and their roughness before and after IP were studied by atomic force microscopy (AFM). AFM images of the surface of TFC membranes without and with a polyamide layer formed via IP with a scan size 5 × 5 µm are presented in Figure 7.

Based on the obtained AFM images the roughness characteristics (values of root-mean-square surface roughness (R_q_) and average surface roughness (R_a_)) of the selective layer surface of the membranes with a thin selective layer based on CS deposited on PAN-2,3,4,5 substrates with and without polyamide layer formed by IP were calculated and presented in Table 4.

The data presented in Table 4 demonstrate that the average roughness of the surface for thin layer from CS on PAN-2,3,4 substrates is practically similar (~1 nm), and for CS/PAN-5 membrane it is slightly higher (1.885 nm). It was found that formation of the polyamide layer via IP on the surface of CS/PAN membrane decreased the roughness of TFC membranes on PAN-2,3,5 substrates, which might indicate the elimination of possible defects of a thin selective layer by the formation of an upper thin polyamide layer after IP. At the same time roughness of CS/PAN-4 membrane practically did not change (1.003 and 1.061 nm, respectively) after the formation of a polyamide selective layer via IP. It should be noted that all membranes have a fairly smooth surface, i.e., no significant changes are observed after polyamide selective layer formation via IP.

**Figure 8:**
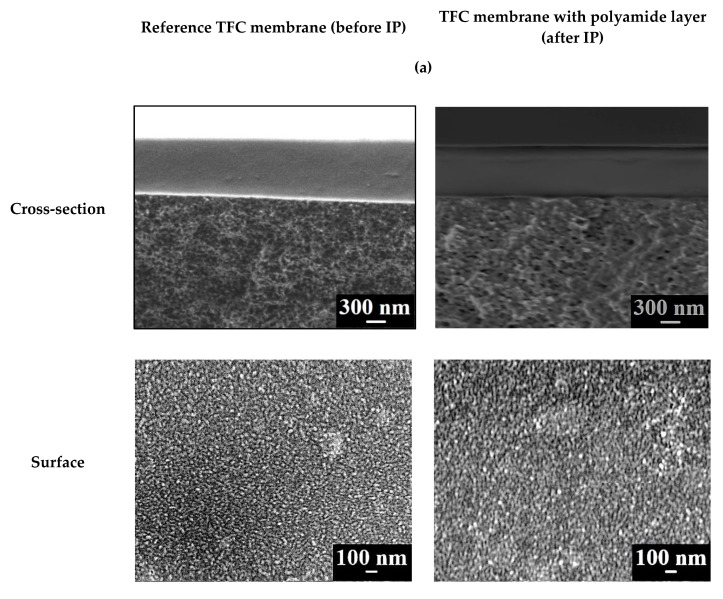

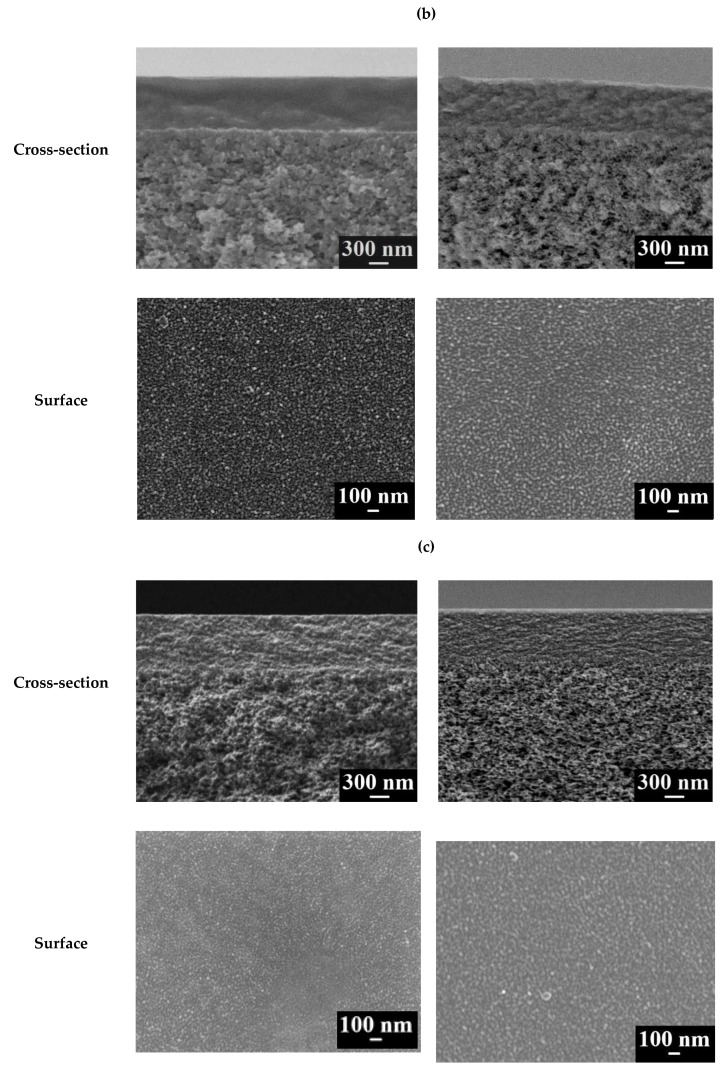

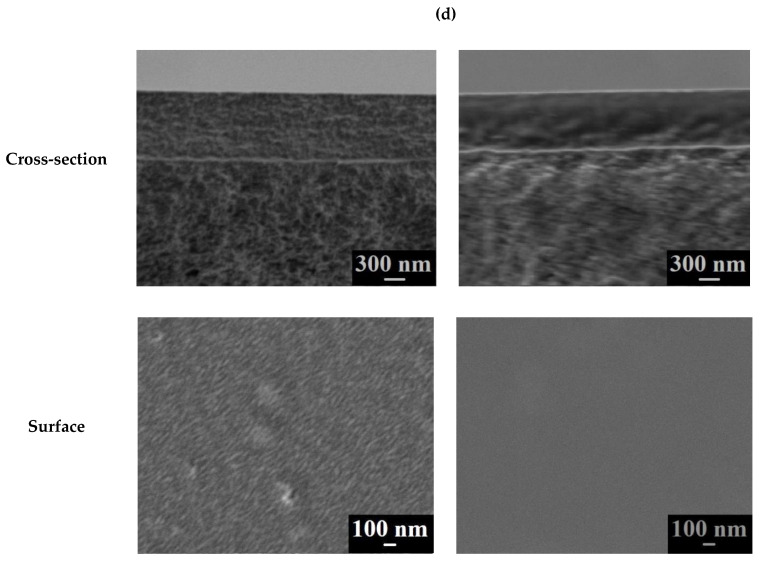
SEM cross-sectional and surface micrographs of the TFC membranes (**a**) CS/PAN-2, (**b**) CS/PAN-3, (**c**) CS/PAN-4, (**d**) CS/PAN-5 with and without polyamide layer formed by IP.

The surface and cross-section morphology of TFC membranes was studied by SEM. SEM micrographs of the surface and the cross-section of TFC membranes with a thin selective layer based on CS deposited of PAN-2,3,4,5 substrates with and without polyamide layer formed by IP are shown in Figure 8.

In all cases the SEM cross-sectional micrographs clearly demonstrate the thin dense CS-based layer with a thickness about ~600–700 nm and the thin layer (second upper layer) on its surface after IP with a thickness about ~10 nm. The surface morphology of membranes with and without the polyamide selective layer formed by IP did not differ significantly and the structure of this layer was similar for all membranes, with the exception of the CS/PAN-5 membrane. Formation of the polyamide thin selective layer on the surface of CS/PAN-5 membrane via IP yielded the formation of a denser uniform surface structure significant decreasing its average roughness (AFM data, Table 4), which could lead to membrane permeation flux decrease compared to the CS/PAN-4 membrane with polyamide layer (Table 5).

It is shown in the next section that the hydrophilic-hydrophobic properties and the surface roughness of the prepared TFC membranes, as well as the pore size and degree of porosity of the selective layer of the selected ultrafiltration membrane (substrate), significantly affect the transport characteristics of the membranes depending on the selected type of PAN membrane (substrate).

### 3.3. Transport Properties of TFC CS-Based Membranes

The transport properties of TFC membranes with a thin dense layer based on chitosan deposited on PAN substrates with different porosity with and without a polyamide layer formed by IP were studied in pervaporation for separation of azeotropic isopropanol/water (88/12 wt.%) mixture. The results of pervaporation experiments of the developed membranes are presented in Table 5.

The data in Table 5 demonstrate high selectivity with respect to water for all membranes with a thin selective layer prepared using 1 wt.% CS solution (96.4–99.9 wt.% water in the permeate depending on the porous substrate used to prepare the TFC membrane). It is also worth noting that changes of permeation flux for the TFC membranes on PAN substrates are in agreement with the data of the measured performance and total porosity of these substrates (PAN-2,3,4,5). The low degree of porosity of the upper (selective) layer of the ultrafiltration membrane (substrate) leads to the formation of a low permeable TFC membrane despite the same thickness of the selective chitosan layer (Figure 8). This is due to the fact that mass transfer of components slows down due to non-porous regions of the selective layer of the ultrafiltration substrate, where some of the penetrant molecules get after diffusion through the selective layer of chitosan. At low porosity of the selective layer of the ultrafiltration substrate, penetrant molecules still need to diffuse through the non-porous regions of the selective layer of the ultrafiltration substrate, which can have a thickness of up to 0.5 μm. Thus, differences in permeation performance of TFC CS/PAN membranes are due to the difference in the pore size, degree of porosity and thickness of the selective layer of PAN substrates. It should be noted that almost the same low permeation flux was observed for supported 1%CS/PAN-2 and 0.5%CS/PAN-2 membranes, which was sharply different from the rest TFC membranes. So the concentration (and therefore also viscosity) of the chitosan solution almost does not affect the permeation flux of TFC membrane based on PAN-2. That indicates that the chitosan-based selective layer is formed in each case in the same way and there is no leakage of the solution into the membrane pores of the selective layer of PAN-2 membrane due to their small size. However, the low porosity of the selective layer of PAN-2 substrate and possibly its large thickness slows down mass transfer through the membrane during pervaporation.

It was shown that the formation of a polyamide layer by IP method on the TFC CS/PAN membrane surface in most cases led to the increase of permeation flux in the pervaporation separation of the azeotropic isopropanol/water mixture with the exception of the 1%CS/PAN-2 membrane. It should be noted that in all cases with the exception 1%CS/PAN-3 and 1%CS/PAN-4 membranes a decrease in the water content in the permeate was observed after formation of a thin polyamide-based layer by IP compared to the unmodified TFC CS/PAN membranes. 1%CS/PAN-4 membrane was found to feature the optimal transport characteristics in pervaporation separation of the azeotropic isopropanol/water mixture: a significant change in the permeation flux after the formation of a polyamide layer by IP from 0.108 to 0.233 kg/(m^2^h) maintaining a high selectivity level (99.9 wt.% water in the permeate). An increase of permeation flux and a decrease of selectivity after the formation of polyamide layer via IP could be associated with a chitosan hydrogen bond system disruption due to the formation of polyamide chains. An aqueous TETA solution in contact with a chitosan layer impregnates the chitosan layer. Despite that chitosan is cross-linked, it is able to swell in water and the amine inserts between the chitosan chains. At the moment of contact of the membrane with an acyl chloride solution in a nonpolar solvent the following may occur: (1) TETA reacts with TMC and moves apart the chitosan chains by the growing polyamide chain, disrupting the dense packing of chitosan chains in the sublayer; (2) hydroxyl or amine chitosan groups react with TMC instead of TETA, polyamide does not form or the growing polyamide chains break, but most important is the disruption of the chitosan hydrogen bond system and a decrease of the packing density in the cross-linked selective chitosan layer. As a result, the polyamide does not form a dense defect-free layer. The polyamide partially breaks off, partially intertwines with chitosan. Consequently, IP often may not lead to increasing the water content in the permeate (especially in the case of 0.5% CS/PAN membranes, where the chitosan layer may be thinner and easier to slightly disrupt due to the implementation of polyamide chains). Also, permeation flux increasing and water content in the permeate decreasing after polyamide layer formation could be associated with increasing of the water sorption on the more hydrophilic and less rough surface of the selective layer, what led to a greater selective layer swelling degree during pervaporation.

To increase the permeability by reducing the selective dense layer thickness, TFC membranes were prepared by deposition of 0.5 wt.% CS solution to the same ultrafiltration membranes (substrates) (Table 5). It was shown that using a lower polymer solution concentration (lower viscosity) led to decreasing in selectivity of TFC membranes and to different changes of permeation flux compared to membranes prepared from 1 wt.% CS solution. Identical permeation performance values were obtained for TFC membranes on PAN-3,4,5 substrates. The formation of a thin polyamide layer by IP method for TFC membranes on PAN-2,3,4,5 substrates led to decreasing in water content in the permeate maintaining the same permeation flux level or a slight increasing in permeability. For example, permeation flux of 0.5%CS/PAN-2 and 0.5%CS/PAN-3 membranes did not change after IP, but for membranes on more porous substrates (PAN-4,5) led to increase of permeability ~1.6 times compared to same reference membranes without polyamide layer.

Thus, the formation of a selective CS layer on PAN substrates by physical adsorption and its subsequent cross-linking (the introduction of MA and heat treatment at 110 °C for 120 min) led to the formation of TFC membranes with high selectivity and relatively high permeation flux, especially for membranes on PAN substrates with higher porosity. Formation of an ultrathin selective layer by IP for TFC membranes with a correctly selected preparation conditions (selection of polymer solution concentration and viscosity, structure of porous membrane-substrate) increased the membrane permeability. Based on the obtained data, it can be concluded that the developed TFC membrane with a thin selective layer formed from 1 wt.% CS solution and deposited on a porous PAN-4 substrate (CS/PAN-4 membrane) with the subsequent heating at 110 °C for 120 min possessed the best transport characteristics after the formation of a thin selective polyamide layer by IP for pervaporation separation of azeotropic isopropanol/water mixture (changes after IP: permeation flux from 0.108 to 0.233 kg/(m^2^h) maintaining a high selectivity of 99.9 wt.% water in the permeate). This membrane was selected for further study.

#### 3.3.1. Transport Properties of the Best TFC Membrane

**Figure 9:**
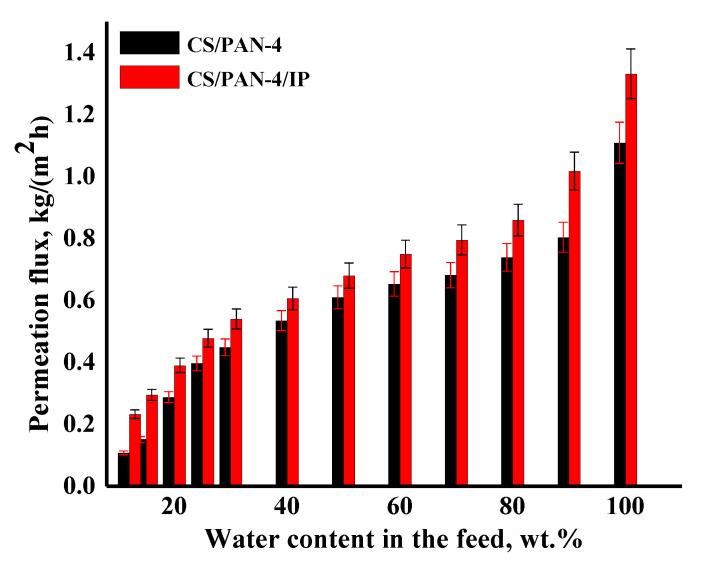
The dependence of the permeation flux on the water content in the feed (12–100 wt.%) in pervaporation dehydration of isopropanol for the reference CS/PAN-4 and CS/PAN-4/IP TFC membranes. Water content in the permeate for all membranes was 99.9 wt.%. The mean accuracy for permeation flux was ±6%.

**Figure 10:**
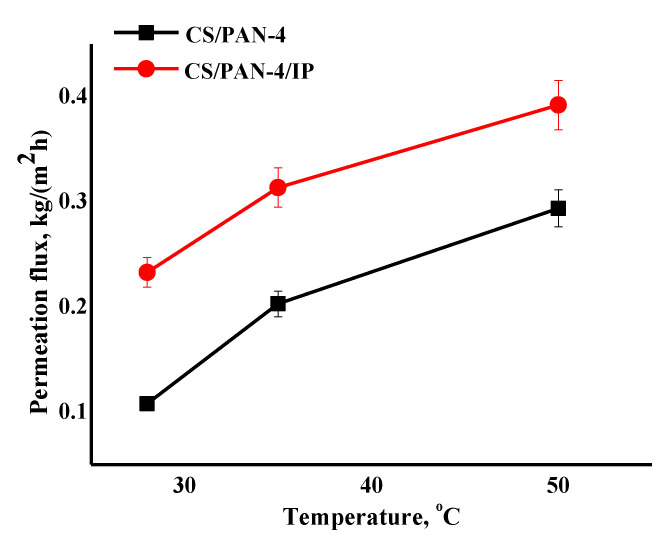
The dependence of the permeation flux on the temperature (28, 35, 50 °C) in pervaporation separation of azeotropic isopropanol/water mixture for the reference CS/PAN-4 and CS/PAN-4/IP TFC membranes. Water content in the permeate for all membranes was 99.9 wt.%. The mean accuracy for permeation flux was ±6%.

Transport properties of the best developed TFC membrane CS/PAN-4 with a polyamide layer (CS/PAN-4/IP membrane) were studied in pervaporation for isopropanol dehydration in a wide concentration range (12–100 wt.% water in the feed) (Figure 9) and for dehydration of the azeotropic isopropanol/water mixture (88/12 wt.%) at various temperatures (28, 35, 50 °C) (Figure 10). The reference membrane CS/PAN-4 was studied for comparison of the transport properties.

It was shown that the CS/PAN-4/IP membrane had higher permeation flux in ~1.2–2.2 times maintaining high selectivity (99.9 wt.% water in the permeate) compared to the reference CS/PAN-4 membrane in pervaporation dehydration of isopropanol. Membranes were also tested for permeability of pure water to evaluate their resistance to water excess and stability of properties. The obtained data demonstrate that the developed membrane with polyamide thin selective layer (CS/PAN-4/IP) is promising to use in industrial dehydration processes where feed may contain a large amount of water.

The data of pervaporation separation of the azeotropic isopropanol/water mixture at different temperatures demonstrate that permeation flux of the reference CS/PAN-4 and CS/PAN-4/IP membranes increases symbatically with the feed temperature, while membranes remain highly selective (99.9 wt.% water in the permeate). The formation of the ultrathin selective layer via IP for the CS/PAN-4 membrane allowed to increase the permeation flux by 1.4–2.2 times maintaining high selectivity level (Figure 10). The properties of the membranes were stable during the pervaporation experiment at elevated temperatures. It also indicates the possibility of using developed TFC membranes in industrial dehydration processes, which are often carried out at elevated temperatures to accelerate the process.

#### 3.3.2. Layer-by-Layer Assembly Application

All prepared membranes had a defect-free thin selective layer. However, to improve the transport characteristics of the CS/PAN-4/IP membrane five bilayers of polyelectrolytes (PEL) were deposited on the membrane surface by the layer-by-layer method.

**Figure 11:**
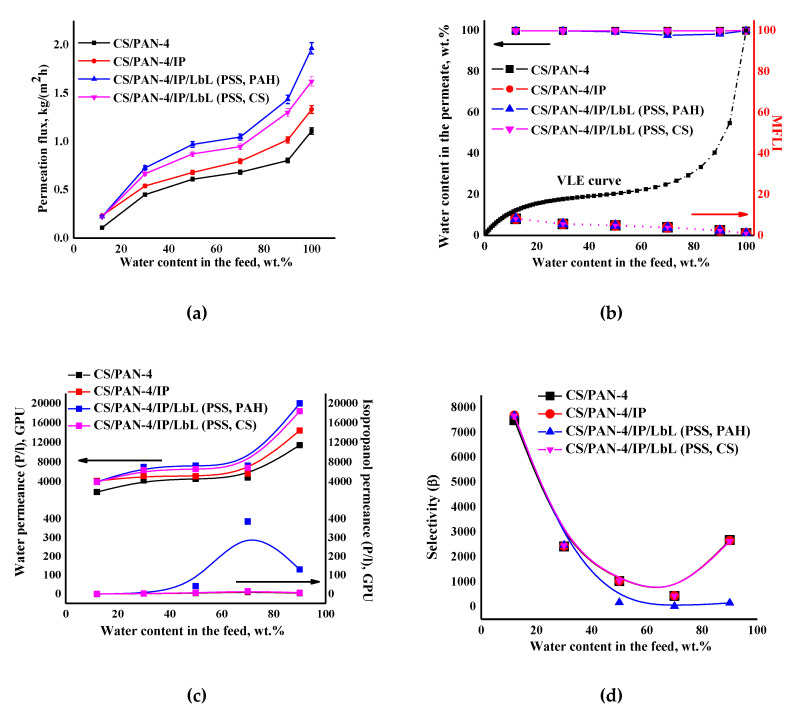
The dependence of (**a**) the permeation flux, (**b**) water content in the permeate and MFLI, (**c**) component permeances and (**d**) selectivity on water content in the feed in pervaporation for separation of isopropanol-water mixture using the reference CS/PAN-4 and unmodified and modified by five bilayers PEL (PSS/PAH or PSS/CS) CS/PAN-4/IP membranes. The vapor–liquid equilibrium (VLE) was calculated using Aspen software. The mean accuracy was <±0.5% for water content in permeate and <±3% for permeation flux.

Transport properties of the modified by PEL layers membranes were studied in pervaporation dehydration of isopropanol in wide concentration range (12–100 wt.% water in the feed) (Figure 11). The pervaporation data of the reference CS/PAN-4 membrane are also presented in Figure 11 for comparison.

It was found that after applying PEL on the membrane surface the permeation flux and water permeance increased compared to a membrane without PEL bilayers due to swelling of polyelectrolytes in water. It was also found that the deposition of five PEL bilayers of PSS/PAH on the CS/PAN-4/IP membrane (for CS/PAN-4/IP/LbL (PSS, PAH) membrane) led to the increase in permeability ~32–47% compared to unmodified CS/PAN-4/IP membrane, while applying of five PEL bilayers of PSS/CS led to the increase in permeation flux ~18–28% for CS/PAN-4/IP/LbL (PSS, CS) membrane. Isopropanol permeance for all membranes was equal except for the CS/PAN-4/IP membrane modified by PSS/PAH (which had a decrease in selective properties).

Figure 11b demonstrates that the curves of water content in the permeate for pervaporation dehydration of isopropanol by using of developed membranes are much higher for the VLE curve for isopropanol-water mixture, which indicates the separation efficiency. Calculated MFLI for developed membranes is also presented in Figure 11b. This characteristic allows to compare pervaporation with the characteristics of elementary flash distillation based only on the separation abilities of these processes. When the MFLI value is even above 1.1, the separation by pervaporation is already preferable rather than distillation. The calculated values of MFLI were relatively the same for all membranes 2.4–8.1, which indicated the high efficiency of the application of membranes for pervaporation dehydration of isopropanol.

It is worth noting that the deposition of five PEL bilayers of PSS/PAH on CS/PAN-4/IP membrane causes the decreasing of membrane selectivity (Figure 11d): decrease of water content in the permeate starts from the separation of 50/50 wt.% isopropanol/water mixture (≥97.7 wt.%) (Figure 11b). At the same time selectivity was maintained at 99.9 wt.% water in the permeate for the entire concentration range of the feed for membrane with PSS/CS polyelectrolyte pair (for CS/PAN-4/IP/LbL (PSS, CS) membrane).

## 4. Conclusions

TFC membranes with a dense selective layer based on chitosan (CS) on the surface of PAN porous substrates (membranes) were prepared.

It was demonstrated that changes in the permeation flux of TFC membranes on PAN substrates in pervaporation isopropanol dehydration were in agreement with the data on the measured permeability and total porosity of PAN substrates. The low porosity of the upper (selective) layer of the ultrafiltration membrane (substrate) led to the formation of a low permeable TFC membrane despite the same thickness of the dense selective CS layer and due to the additional mass transfer resistance of the upper selective layer of the ultrafiltration membrane. The differences in permeation performance of the TFC CS/PAN membranes were due to the differences in the pore size, degree of porosity and thickness of the selective layer of the ultrafiltration membrane (substrate).

It was found that the formation of an additional polyamide (PA) selective layer by the interfacial polymerization (IP) on the surface of a dense selective CS layer, as a rule, yielded the hydrophilization of the selective layer surface of the membrane (water contact angle decreased). Moreover, it decreased surface roughness, increased permeation flux and decreased water content in the permeate in the pervaporation separation of the azeotropic isopropanol-water mixture. The TFC membrane with a selective CS layer deposited on the PAN-4 substrate possessed optimal transport characteristics in pervaporation dehydration of isopropanol (for azeotropic mixture). A significant change of permeation flux after the formation of thin polyamide layer via IP from 0.108 to 0.233 kg/(m^2^h) maintaining a high selectivity level (99.9 wt.% water in the permeate) was found.

Additional layer-by-layer deposition of five bilayers of PSS/PAH or PSS/CS polyelectrolyte pairs on TFC membrane with a selective PA/CS layers formed on the PAN-4 substrate led to the increase in membrane permeation flux ~2 times compared to the reference CS/PAN-4 membrane for pervaporation dehydration of isopropanol. It was due to the additional swelling of the polyelectrolyte layers in water. Moreover, the application of PSS/CS allowed to maintain high selectivity (99.9 wt.% water in the permeate) for the entire concentration range of the feed. Thus, the TFC membrane with hierarchical structure and ultrathin selective layers was developed which demonstrated the optimal performance in pervaporation dehydration of isopropanol (12–90 wt.%): 0.22–1.30 kg/(m^2^h), 99.9 wt.% water in the permeate.

## Figures and Tables

**Table 1:** Roughness characteristics and water contact angles for porous PAN membranes (substrates). The mean accuracy was <±2% for roughness characteristics and ±2° for contact angle.

Membrane	Ra, nm	Rq, nm	Water Contact Angle, °
PAN-2	2.000	2.501	41
PAN-3	2.562	3.260	41
PAN-4	2.607	3.258	41
PAN-5	2.593	3.271	41

**Table 2:** PAN membrane performance in ultrafiltration of PVP K-30 and BSA solutions and total membrane porosity.

Membrane	Pure Water Flux, L/(m^2^·h)	Flux, L/(m^2^·h)		R, %		FRR, % (BSA)	Total Porosity, %
		PVP K-30	BSA	PVP K-30	BSA		
PAN-2	20–40	10	23	>99	96	100	41
PAN-3	72–100	30	80	90	98	97	64
PAN-4	130–160	60	101	75	95	79	73
PAN-5	180–200	40	110	67	95	78	94

**Table 3:** PAN membrane performance in ultrafiltration of PVP K-30 and BSA solutions and total membrane porosity. The mean accuracy for contact angle was ±2°.

TFC Membranes	Contact Angle of Water, °	
	Reference TFC Membrane	TFC Membrane with Polyamide Layer
CS/PAN-2	76	59
CS/PAN-3	77	66
CS/PAN-4	74	68
CS/PAN-5	72	65

**Table 4:** CS-based membrane roughness characteristics. The mean accuracy for roughness characteristics was <±2%.

TFC Membranes	Reference TFC Membrane		TFC Membrane with Polyamide Layer	
	Ra, nm	Rq, nm	Ra, nm	Rq, nm
CS/PAN-2	1.035	1.356	0.994	1.384
CS/PAN-3	1.155	1.532	0.719	0.985
CS/PAN-4	1.003	1.293	1.061	1.444
CS/PAN-5	1.885	2.570	0.963	1.314

**Table 5:** Transport characteristics of TFC CS-based membranes with and without polyamide layer formed by IP in separation of azeotropic isopropanol/water (88/12 wt.%) mixture. The mean accuracy was <±1% for water content in permeate and <±8% for permeation flux.

TFC Membranes	Reference TFC Membrane		TFC Membrane with Polyamide Layer	
	Permeation Flux kg/(m^2^·h)	Water Content in Permeate, wt.%	Permeation Flux kg/(m^2^·h)	Water Content in Permeate, wt.%
1%CS/PAN-2	0.025	99.9	0.022	98.7
1%CS/PAN-3	0.090	97.5	0.130	99.9
1%CS/PAN-4	0.108	99.9	0.233	99.9
1%CS/PAN-5	0.130	96.4	0.160	92.9
0.5%CS/PAN-2	0.023	99.6	0.022	97.0
0.5%CS/PAN-3	0.120	96.5	0.120	95.3
0.5%CS/PAN-4	0.120	99.4	0.200	98.6
0.5%CS/PAN-5	0.120	95.9	0.190	94.6

## Abbreviation

Alg	Alginates
AFM	Atomic force microscopy
BSA	Bovine serum albumin
CS	Chitosan
IR	Infrared spectroscopy
IP	Interfacial polymerization
LbL	Layer-by-layer assembly
MA	Maleic acid
DMF	N,N-dimethylformamide
NIPS	Non-solvent induced phase inversion
PAH	Poly (allylamine hydrochloride)
PSS	Poly (sodium 4-styrenesulfonate)
PAN	Polyacrylonitrile
PEL	Polyelectrolyte
PVA	Polyvinyl alcohol
PVP	Polyvinylpyrrolidone
SEM	Scanning electron microscopy
TFC	Thin-film composite membrane
TETA	Triethylenetetramine
TMC	Trimesoyl chloride
